# Comparative Toxic Effects of Manufactured Nanoparticles and Atmospheric Particulate Matter in Human Lung Epithelial Cells

**DOI:** 10.3390/ijerph18010022

**Published:** 2020-12-22

**Authors:** Yun Wu, Mei Wang, Shaojuan Luo, Yunfeng Gu, Dongyang Nie, Zhiyang Xu, Yue Wu, Mindong Chen, Xinlei Ge

**Affiliations:** 1 Jiangsu Key Laboratory of Atmospheric Environment Monitoring and Pollution Control (AEMPC), Collaborative Innovation Center of Atmospheric Environment and Equipment Technology (AEET), School of Environmental Science and Engineering, Nanjing University of Information Science & Technology (NUIST), Nanjing 210044, China; wuyun@nuist.edu.cn (Y.W.); meiwang@nuist.edu.cn (M.W.); 1528805335a@gmail.com (Y.G.); 201883310062@nuist.edu.cn (Z.X.); 201883310072@nuist.edu.cn (Y.W.); chenmd@nuist.edu.cn (M.C.); 2 School of Chemical Engineering and Light Industry, Guangdong University of Technology, Guangzhou 510006, China; kesjluo@gdut.edu.cn; 3 School of Atmospheric Sciences, Nanjing University, Nanjing 210023, China; dynie@nju.edu.cn

**Keywords:** atmospheric particulate matter, nanoparticles, A549 cells, cytotoxicity

## Abstract

Although nanoparticles (NPs) have been used as simplified atmospheric particulate matter (PM) models, little experimental evidence is available to support such simulations. In this study, we comparatively assessed the toxic effects of PM and typical NPs (four carbonaceous NPs with different morphologies, metal NPs of Fe, Al, and Ti, as well as SiO_2_ NPs) on human lung epithelial A549 cells. The EC50 value of PM evaluated by cell viability assay was 148.7 μg/mL, closest to that of SiO_2_ NPs, between the values of carbonaceous NPs and metal NPs. All particles caused varying degrees of reactive oxygen species (ROS) generation and adenosine triphosphate (ATP) suppression. TiO_2_ NPs showed similar performance with PM in inducing ROS production (*p* < 0.05). Small variations between two carbonaceous NPs (graphene oxides and graphenes) and PM were also observed at 50 μg/mL. Similarly, there was no significant difference in ATP inhibition between carbonaceous NPs and PM, while markedly different effects were caused by SiO_2_ NP and TiO_2_ NP exposure. Our results indicated that carbonaceous NPs could be served as potential surrogates for urban PM. The identification of PM model may help us further explore the specific roles and mechanisms of various components in PM.

## 1. Introduction

Atmospheric particulate matter (PM) is a major environmental pollutant endangering human health. It can penetrate and deposit deep inside the lungs, initiate a local inflammation or attack remote tissues by translocated particles [1,2]. Epidemiological and toxicological studies have demonstrated that chronic exposure to PM may induce or aggravate a variety of diseases including asthma, stroke, ischemic heart disease, chronic obstructive pulmonary disease, and lung cancer [3,4,5]. PM is an extremely complicated mixture of black carbon, sulfate, nitrate, ammonium, metals, and many organic compounds [6,7]. PM also varies greatly in particle size and surface property. Therefore, although there are numerous studies on the toxic effects of PM, its exact toxic components and the underlying mechanisms are largely unknown.

Meanwhile, nanotechnology is a rapidly growing area with a number of emerging materials. The widespread application of nanoparticles (NPs) in industrial and consumer products raises important concerns about human exposure and health risk [8,9]. Although oral ingestion and dermal absorption are important routes of NPs entry into the body, inhalation exposure is also considered as a common route and has received much attention [10]. NPs can be deposited in the lungs and a part of them can spread into the blood circulation, causing harmful effects on multiple organs and systems. At present, the action mechanisms of NPs include (but are not limited to): generation of reactive oxygen species (ROS) and proinflammatory cytokines, impairment of phagocytosis and biological barriers, alteration of cell cycle regulation and metabolic activity [8,11]. Similarly, there are great variations in shape, composition, and property of NPs, which complicate their toxicity.

Theoretically, NPs with clearer structures and simpler components might be considered as suitable models for investigating the key toxic components and their associations in PM, since the microscopic structures of most PM are aggregates consisting of NPs ranging in size from 40 to 70 nm [12]. For example, Soto et al. [13] used the commercial carbonaceous particles as surrogate materials for combustion PM. Jia et al. [5] used carbon nanocarriers with adsorbed pollutants to study the specific roles of various pollutants in PM_2.5_, and indicated that Cr(VI) and Pb^2+^ contributed to PM_2.5_-induced reduction in cell viability. However, to the best of our knowledge, there is little systematic study on the comparative toxicity of NPs and PM, let alone the underlying mechanisms (similar or different). Therefore, little experimental evidence is available to support such model simplification.

In the present study, we compared the toxic effects of PM and NPs on human lung epithelial A549 cells (target models). Considering the major components of PM, eight manufactured NPs with different compositions, structures, and dimensions were selected, including aluminum oxide nanoparticles (Al_2_O_3_ NPs), titanium dioxide nanoparticles (TiO_2_ NPs), silicon dioxide nanoparticles (SiO_2_ NPs), iron oxide nanoparticles (FeOx NPs), multi-walled carbon nanotubes (MWCNTs), black carbons (BCs), graphene oxides (GOs), and graphenes. We examined the physicochemical properties of different particles and their toxic effects on cell viability, ROS generation, and adenosine triphosphate (ATP) synthesis, seeking to explore which NPs could be used as suitable surrogates for urban PM, if any.

## 2. Materials and Methods

### 2.1. Test Substances

Al_2_O_3_ NPs, TiO_2_ NPs, SiO_2_ NPs, MWCNTs, BCs, and GOs were purchased from XFNANO Materials Tech Co., Ltd. (Nanjing, China), and graphenes were provided by Strem Chemicals, Inc (Newburyport, MA, USA). FeOx NPs were synthesized using iron (III) chloride hexahydrate (FeCl_3_·6H_2_O) according to a mechanochemical method with a few modifications (three times KCl addition) [14]. The urban particulate matter sample (SRM 1648a), obtained from the National Institute of Standards and Technology (NIST), was used as a standard for comparative toxicity evaluation between PM and NPs.

All particles were subjected to the same procedure for characterization. After dispersion in the culture medium by ultrasonication (5 min), number-weighted hydrodynamic diameters and zeta potentials were characterized by dynamic light scattering (DLS, Brookhaven NanoBrook 90Plus PALS, Holtsville, NY, USA) and zeta potential analyzer (Malvern, Zetasizer Nano ZS90, Worcestershire, UK), respectively. The shape, size, and agglomeration status of NP/PM samples were then determined using a field emission scanning electron microscope (FESEM, Hitachi SU8220, Tokyo, Japan).

### 2.2. Cell Culture and Exposure Conditions

The human lung epithelial cell line A549 was obtained from Nanjing University of Chinese Medicine. The cells were cultured in Minimum Essential Medium (MEM, KeyGEN BioTECH, Nanjing, China) supplemented with 10% fetal bovine serum (FBS, Gibco, Melbourne, Australia), at 37 °C in a humidified atmosphere with 5% CO_2_. Phosphate Buffer Saline (PBS) and trypsin (0.25%), purchased from KeyGEN BioTECH, Nanjing, China, were also used during cell subculture. At 80% confluence, A549 cells were seeded in 96-well plates (5 × 10^3^ cells per well) or 6-well plates (5 × 10^5^ cells per well). After incubation overnight, cells were washed with PBS and treated with different concentrations of particles. Dimethyl sulfoxide (DMSO) was used to facilitate the solution of particles, and its final concentrations (0.1%) in the exposure medium showed no measurable effect on our tested endpoints. Stock solutions of particles were diluted with MEM, and ultrasonicated to ensure better dispersion before use. Based on the results of our preliminary experiments, six concentrations of each stimulus were selected for cell viability assay to calculate the concentration that produced 50% of maximal effect (EC50). The nominal concentrations were: Al_2_O_3_ NPs (0, 50, 150, 200, 350 and 500 μg/mL), TiO_2_ NPs (0, 50, 120, 180, 300 and 500 μg/mL), SiO_2_ NPs (0, 50, 150, 180, 300 and 500 μg/mL), FeOx NPs (0, 0.5, 5, 10, 40 and 100 μg/mL), MWCNTs (0, 20, 40, 100, 150 and 300 μg/mL), BCs (0, 10, 50, 120, 150 and 300 μg/mL), GOs (0, 1, 30, 50, 100 and 125 μg/mL), graphenes (0, 10, 50, 80, 100 and 150 μg/mL), and SRM 1648a (0, 10, 60, 100, 120 and 150 μg/mL). Further, an identical concentration of 50 μg/mL (toxic concentration) and the corresponding EC50 (obtained from cell viability assay) were chosen for ROS and ATP detection.

### 2.3. Cell Viability Assay

Contrasting effects of PM and NPs on A549 cells were first measured by CCK-8 assay (Cell counting kit-8, Beyotime, Shanghai, China) according to the manufacturer’s guidelines. Briefly, cells were seeded in 96-well plates, incubated overnight, and then exposed to different levels of particles for 24 h. Afterward, 10 μL of CCK-8 solution was added to each well, and the plates were incubated at 37 °C for another 1 h. The absorbance at 450 nm was monitored with a multifunction microplate reader (Molecular Devices SpectraMax-iD3, Sunnyvale, CA, USA). In addition, the interferences of particles on absorbance were reduced by subtracting the absorbance of exposed cells in the exposure medium at 600 nm [15]. Cell viability was calculated by the following equation: cell viability (%) = (A − B)/(C − B) × 100%, where A is the absorbance of the treated cells; B is the absorbance of blank controls (culture medium only); and C is the absorbance of the untreated cells. These experiments were performed in sextuplicate and repeated at least twice.

### 2.4. ROS Measurement

A peroxide sensitive fluorescent probe, 2′,7′-dichlorofluorescein diacetate (DCFH-DA, Sigma, USA), was used for the assessment of intracellular ROS production. A549 cells were seeded in 6-well plates and incubated overnight. The culture medium was then carefully replaced by the exposure medium containing the designated concentrations of stimuli (0, 50 μg/mL and the calculated EC50 for each stimulus). After 24 h of exposure, the cells were washed with PBS twice, and incubated with 1 mL of DCFH-DA (20 μM) for 25 min at 37 °C. Thereafter, the cells were washed again with PBS, detached with trypsin, and collected by centrifugation (400× *g*, 2 min). For each sample, at least 5000 cells were analyzed for the corresponding fluorescence with a flow cytometer (Beckman CytoFLEX FCM, Brea, CA, USA) at 488 nm excitation and 525 nm emission. ROS content was expressed as the relative fluorescence of treated cells relative to that of untreated cells. These experiments were performed in triplicate and repeated at least twice.

### 2.5. ATP Measurement

Effects of NPs and PM on cellular ATP concentrations were detected by the luciferin-luciferase method [16] following the protocol of ATP assay kit (Beyotime, Shanghai, China). Confluent A549 cells were treated with three concentrations of particles (0, 50 μg/mL and the calculated EC50 for each stimulus) in 6-well plates for 24 h. After washing twice with PBS, 200 μL of lysis buffer was added to facilitate ATP release. The suspension was then centrifuged at 400× *g* for 5 min (4 °C), and 20 μL of the supernatant was mixed with an ATP detection buffer. Luminescence analysis was carried out with a multifunction microplate reader. ATP levels were calculated using the standard curve prepared in a similar manner (10 nM to 3 μM). These experiments were performed in triplicate and repeated at least twice.

### 2.6. Statistical Analysis

The results were presented as the mean ± standard deviation (SD). Linear interpolation method (ICPIN software, version 2.0, USEPA) was used to calculate the EC50 in the CCK-8 assay. Differences among treatments were analyzed using one-way analysis of variance (ANOVA), with the Duncan post hoc test or with Dunnett’s test where applicable (SPSS version 26.0, IBM company, Armonk, NY, USA). Statistical significance was accepted when *p* < 0.05.

## 3. Results

### 3.1. Characterization of Test Particles

**Figure 1:**
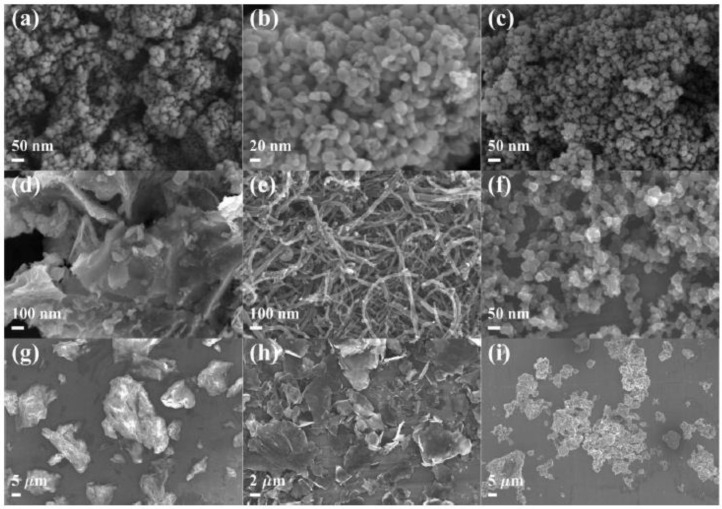
Typical field emission scanning electron microscope (FESEM) images of aluminum oxide nanoparticles (Al_2_O_3_ NPs) (**a**), titanium dioxide nanoparticles (TiO_2_ NPs) (**b**), silicon dioxide nanoparticles (SiO_2_ NPs) (**c**), iron oxide nanoparticles (FeOx NPs) (**d**), multi-walled carbon nanotubes (MWCNTs) (**e**), black carbons (BCs) (**f**), graphene oxides (GOs) (**g**), graphenes (**h**), and particulate matter (PM) (**i**).

PM and NPs with different physicochemical properties were studied in a comparative way. FESEM images of these nine particles exhibited considerable diversity in their morphologies and agglomeration (Figure 1). Al_2_O_3_ NPs, TiO_2_ NPs, SiO_2_ NPs, and BCs were near-spherical ranging in size from 10 to 60 nm in diameter, and tended to aggregate to hundreds to thousands of nm. Synthesized FeOx NPs presented a nano-plate structure, while MWCNTs revealed the expected tubular structure of 10 to 50 nm in diameter and 0.5 to 2.0 μm in length (data from manufacturers). Both GOs and graphenes showed a sheet-like morphology; however, different brightness intensities were observed on the GO and graphene flakes, indicating their different thicknesses and surface wrinkles. The PM sample showed an irregular morphology due to its complex composition, with a wide particle size range of 1 to 26 μm.

Table 1 shows the hydrodynamic diameters (0 h and 48 h) and zeta potentials of NP and PM samples. The mean agglomerate diameters (0 h) followed the order of SiO_2_ NPs < BCs < TiO_2_ NPs < FeOx NPs < Al_2_O_3_ NPs < MWCNTs < GOs < PM < graphenes, and remained relatively constant over 48 h of interaction (*p* > 0.05), with a few exceptions (TiO_2_ NPs, SiO_2_ NPs, and GOs). The results of zeta potentials showed that all particles were negatively charged at pH 7.4 (between −18.8 ± 2.3 mV and −27.6 ± 1.6 mV).

### 3.2. Cytotoxicity and Intracellular Response

**Figure 2:**
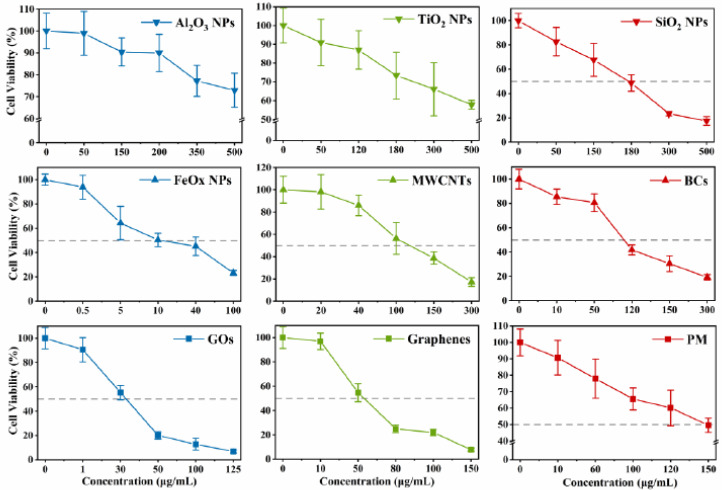
The changes in the viability of A549 cells exposed to different NPs and PM. Data are mean ± SD (n = 12).

**Figure 3:**
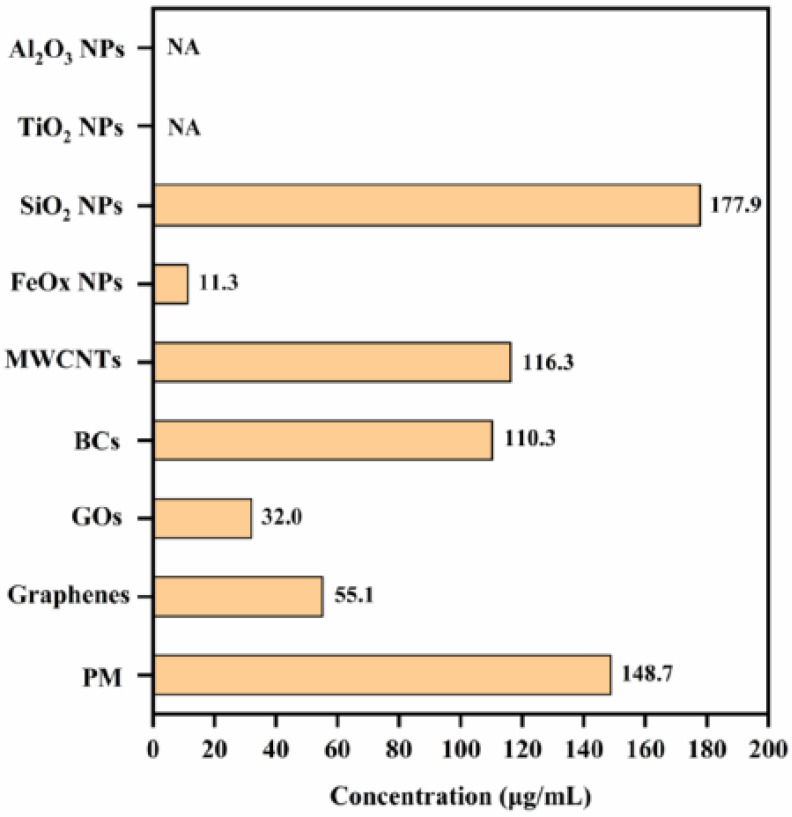
The calculated EC50 values of nine test particles. “NA” means not available.

The changes in the viability of A549 cells exposed to different NP/PM concentrations (up to 500 μg/mL) are shown in Figure 2, and the calculated EC50 values for all test particles are shown in Figure 3. Generally, NP/PM exposure decreased A549 cell viability in a dose-dependent manner with different cytotoxicity profiles. The EC50 values of Al_2_O_3_ NPs and TiO_2_ NPs were not available within the concentration ranges tested. When the concentration of Al_2_O_3_ NPs and TiO_2_ NPs was below 350 and 300 μg/mL, respectively, no significant decrease was observed in cell viability, indicating their relatively low cytotoxicity. Based on the obtained EC50 values, SiO_2_ NPs exhibited the lowest cytotoxicity, while FeOx NPs showed the highest cytotoxicity. The variation in EC50 among the four carbonaceous NPs was large (MWCNTs: 116.3 μg/mL; BCs: 110.3 μg/mL; GOs: 32.0 μg/mL; and graphenes: 55.1 μg/mL), which may be related to their different chemical compositions and structural features. The EC50 value of PM was 148.7 μg/mL, somewhere in between the carbonaceous NPs and metal NPs. Significant differences were observed at 50 µg/mL of SiO_2_ NPs, FeOx NPs, BCs, GOs, and graphenes compared to controls (*p* < 0.05). Therefore, we further compared the toxic effects between NPs and PM at two concentrations: the same concentration (50 µg/mL) and different concentrations but having similar effects in cell viability assay (EC50), in the following experiments.

**Figure 4:**
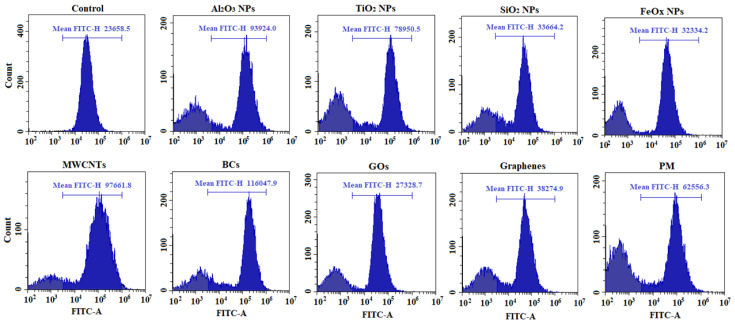
Representative histograms of flow cytometric analysis of reactive oxygen species (ROS) production in A549 cells with or without NP/PM exposure. The exposure concentrations (EC50) were 177.9 μg/mL for SiO_2_ NPs, 11.3 μg/mL for FeOx NPs, 116.3 μg/mL for MWCNTs, 110.3 μg/mL for BCs, 32.0 μg/mL for GOs, 55.1 μg/mL for graphenes, and 148.7 μg/mL for PM. The concentration of 500 μg/mL was used here for Al_2_O_3_ NPs and TiO_2_ NPs. Mean FITC-H values (yellow-green fluorescence) were used to calculate the relative ROS levels.

**Figure 5:**
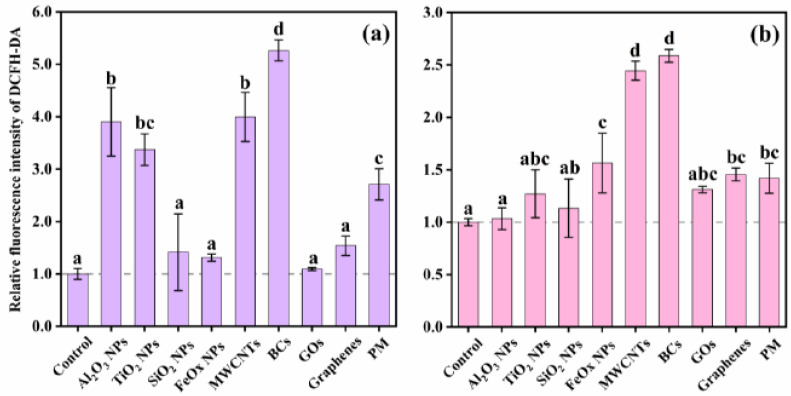
Generation of reactive oxygen species (ROS) in A549 cells upon exposure to NPs and PM at the calculated EC50 (**a**) and a constant toxic concentration of 50 μg/mL (**b**). The EC50 values were 177.9 μg/mL for SiO_2_ NPs, 11.3 μg/mL for FeOx NPs, 116.3 μg/mL for MWCNTs, 110.3 μg/mL for BCs, 32.0 μg/mL for GOs, 55.1 μg/mL for graphenes, and 148.7 μg/mL for PM. The concentration of 500 μg/mL was used here for Al_2_O_3_ NPs and TiO_2_ NPs. Values for each stimulus sharing no common letters (a, b, c and d) were significantly different in the same experiment (*p* < 0.05, Duncan post hoc multiple comparisons). Data are mean ± SD (n = 6).

Representative histograms of flow cytometric analysis of ROS production in A549 cells with or without NP/PM exposure are shown in Figure 4, and relative ROS induction at the EC50 for each stimulus is shown in Figure 5a. Although cell viability was reduced to a similar level (50%), ROS production varied greatly and exhibited the following sequence: BCs > MWCNTs > Al_2_O_3_ NPs > TiO_2_ NPs > PM > graphenes > SiO_2_ NPs > FeOx NPs > GOs. Exposure to the former five led to a statistically significant increase in the intracellular ROS level in comparison to controls, while exposure to the latter four showed comparable or slightly higher ROS generation than those of the controls. Further, comparative effects of nine particles on ROS generation were assessed at a constant concentration of 50 μg/mL (Figure 5b). Similarly, BCs and MWCNTs induced the most amount of ROS (2.6-fold and 2.4-fold, respectively), indicating their strong ability to produce oxidative stress. Additionally, the minimum ROS levels were found in A549 cells upon exposure to Al_2_O_3_ NPs, SiO_2_ NPs, and TiO_2_ NPs (*p* < 0.05) at the designated concentration. Different from the results obtained at the EC50, ROS production induced by Al_2_O_3_ NPs and TiO_2_ NPs showed no significant difference with respect to untreated controls, which may be related to the dramatic variation in exposure concentration (500 μg/mL vs. 50 μg/mL). FeOx NPs, graphenes, GOs, and PM caused comparable degrees of oxidative stress response (1.3–1.6 fold). When we combined these two figures, we found that intracellular ROS production induced by PM was similar to that induced by TiO_2_ NPs. Small variations between two carbonaceous nanomaterials (GOs and graphenes) and PM were observed at the EC50 (1.1–2.7 fold), while these variations were insignificant at the concentration of 50 μg/mL (*p* < 0.05), which might be explained by the fact that carbonaceous particles are major components in PM.

**Figure 6:**
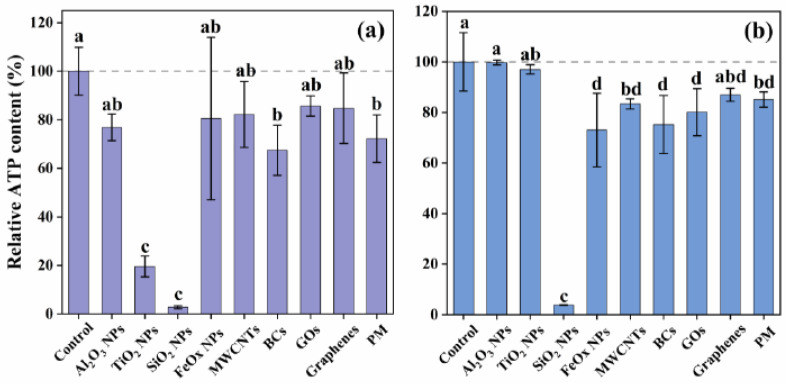
Changes in adenosine triphosphate (ATP) levels upon exposure to NPs and PM at the calculated EC50 (**a**) and a constant toxic concentration of 50 μg/mL (**b**). The EC50 values were 177.9 μg/mL for SiO_2_ NPs, 11.3 μg/mL for FeOx NPs, 116.3 μg/mL for MWCNTs, 110.3 μg/mL for BCs, 32.0 μg/mL for GOs, 55.1 μg/mL for graphenes, and 148.7 μg/mL for PM. The concentration of 500 μg/mL was used here for Al_2_O_3_ NPs and TiO_2_ NPs. Values for each stimulus sharing no common letters (a, b, c and d) were significantly different in the same experiment (*p* < 0.05, Duncan post hoc multiple comparisons). Data are mean ± SD (n = 6).

Mitochondria are one of the major target organelles for excessive ROS. Structural and functional impairment of mitochondria could result in metabolic arrest, and then affect ATP synthesis. Therefore, we determined the levels of ATP in NP/PM-treated cells (Figure 6). As expected, almost all test particles showed damage to mitochondrial function, with varying degrees of ATP inhibition. Particularly, SiO_2_ NPs had the strongest effects on ATP synthesis (2.8 ± 0.6% at the EC50 and 3.9 ± 0.1% at 50 μg/mL), although these particles did not induce the largest amount of intracellular ROS, indicating that other damage mechanisms might be involved. Consistent with the results of ROS measurement, effects on ATP content inhibited by Al_2_O_3_ NPs and TiO_2_ NPs occurred in a dose-dependent manner that particles at 500 μg/mL revealed a more pronounced inhibition than at 50 μg/mL. There was no statistical difference in the intracellular ATP content between A549 cells treated with carbonaceous particles and PM (EC50: 82.2 ± 13.6% for MWCNTs, 67.5 ± 10.3% for BCs, 85.7 ± 4.2% for GOs, 84.8 ± 14.5% for graphenes, 72.2 ± 9.8% for PM; 50 μg/mL: 83.4 ± 2.0% for MWCNTs, 75.2 ± 11.5% for BCs, 80.1 ± 9.4% for GOs, 87.0 ± 2.6% for graphenes, 85.1 ± 3.0% for PM). The results obtained at these two concentrations again implied their comparable toxic effects.

## 4. Discussion

PM can penetrate deep into the respiratory tracts, induce epithelial cell damage and disrupt normal lung function [17]. Consistent with our previous study [18], PM exhibited marked toxicity to A549 cells with an EC50 of 148.7 µg/mL. However, the chemical composition, size distribution, and surface structure of PM are extremely complex, not only related to the sources of PM but also to the influences of environmental conditions [5,19]. Consequently, there was notable variation in the EC50 values of ambient PM (in the range of tens to hundreds µg/mL) [5,20]. Moreover, these variations make it difficult to understand the mechanisms of PM-induced toxicity. Simplified models were then proposed to promote the study of PM toxicity.

Chemical-induced toxicity depends firstly on the substance contained therein; thus, surrogate models should ideally have similar constituents as ambient PM. Numerous studies have analyzed the principal components of PM: carbonaceous particles were the dominant component in urban areas characterized by high traffic intensity and combustion emission [21]; silicon was a major component of PM core originated from soil dust and construction activity [22]; metal particles represented another important component of PM, and iron oxide particles were the most abundant species [23]. Therefore, four carbonaceous particles with different morphologies, metal NPs of Fe, Al and Ti, as well as SiO_2_ NPs, were selected as PM candidates in this study. According to the EC50 values from cell viability assay, the toxic effects of PM were most closely related to those of SiO_2_ NPs (177.9 μg/mL), MWCNTs (116.3 μg/mL), and BCs (110.3 μg/mL), followed by other carbonaceous particles. These results indicated that SiO_2_ NPs or carbonaceous NPs might be used as PM cores to study their toxicity. And more attention should be paid to the PM core itself, which might contribute to the majority of PM-induced toxicity directly. Further, it has been reported that metal components in PM contributed to approximately 7.3–22.9% of PM-induced toxicity [18,24], while organic components (e.g., polycyclic aromatic hydrocarbon) might not play important roles in cell toxicity [13]. Because the chemical compositions of PM are extremely complex, PM models adsorbed with varying degrees of substances could facilitate our understanding of their toxic contributions and mechanisms.

Both PM and NPs can affect membrane integrity through direct mechanical action due to their irregular size and/or shape [25,26]. Observed reductions in cell viability may be partly explained by particle damage to cell membranes. In addition, oxidative stress is now well accepted as a major mechanism for the toxic effects of inhaled PM and NPs [8,27]. For one thing, particles can directly induce ROS generation [28]; for another, cellular responses also contribute to oxidative stress upon exposure to particles [29]. Then, elevated ROS levels can cause DNA, protein and membrane damage, leading eventually to cell death/apoptosis [30], which explains part of the suppression of cell viability. Furthermore, particles with multiple sizes and chemical compositions have been shown to be preferentially located in mitochondria. Mechanical action together with oxidative stress result in the destruction of mitochondria [31,32]. Impairment of mitochondrial structure and function initiates a series of cellular reactions, including disruption of metabolic activities and inhibition of ATP synthesis [32]. As expected, our results showed that all nine particles at two concentrations reduced cell viability, increased ROS production, and decreased ATP content to different degrees. ROS generation induced by PM was similar to that induced by TiO_2_ NPs, as well as two carbonaceous NPs (GOs and graphenes), intracellular ATP content showed no significant difference between carbonaceous NP treatments and PM treatments, again implying their comparable toxic actions. Although SiO_2_ NPs had similar effects on cell viability compared with PM, they showed contradictory results in ATP inhibition, probably because SiO_2_ NPs directly caused mitochondrial damage and thus dramatically decreased intracellular ATP content [33]. When we combined our results, we could conclude that carbonaceous NPs (including BCs, MWCNTs, GOs, and graphenes) had similar cytotoxicity and action mechanisms with PM, indicating their potentials as surrogate models for PM.

Apart from material compositions, physicochemical properties of particles have been considered as important factors governing their toxic effects, such as size distribution [34], particle shape [35], aggregation state [36], and surface charge [37]. Generally, NPs with smaller sizes and larger surface areas are expected to have higher toxicity [38]. The same is true for atmospheric particles, despite the fact that finer particles contained higher amounts of inorganic and organic pollutants [19]. Although spherical particles are preferentially taken up into cells, the geometry of non-spherical particles have been hypothesized to cause higher toxicity because of their damage to cell membrane and cellular structure [39,40]. An observational study in urban areas characterized carbonaceous PM and reported two different morphologies: chains of spherules with dimensions up to 2 μm and aggregated spherules with diameters between 360 nm and 4 μm [12]. Irregular-shaped plates ranging in size from 1 to 26 μm were also observed in this study. PM is very different in size and shape. Thus, it is desirable to mimic the toxicity of PM using two or more carbonaceous NPs. Carbonaceous NPs used in this study (except for BCs) were micrometer-scale particles in at least one dimension, and they might agglomerate in solution. Therefore, hydrodynamic diameters of PM were comparable to those of carbonaceous NPs. In addition, both PM and NPs were negatively-charged, which could be absorbed onto the positively-charged lipids in cell membrane [41]. Considering the complexity of PM properties and their similarity with carbonaceous NPs, we hypothesize that a mixture of carbonaceous NPs may be a more suitable surrogate for PM, yet the detailed formula deserves further investigation.

## 5. Conclusions

Our study provided experimental evidences that carbonaceous NPs could be used as surrogate models to study the toxic mechanisms of PM. NPs and PM reduced cell viability, increased ROS production, and decreased ATP content to varying extents. The reduction in cell viability caused by PM was almost equivalent to that caused by SiO_2_ NPs, followed by four carbonaceous NPs. Small variations in ROS generation and ATP inhibition were also observed between carbonaceous NPs and PM. However, SiO_2_ NPs had the strongest negative effects on ATP synthesis, which may be explained by their direct damage to mitochondria. These results indicated that PM-induced toxicity depended greatly on its components. Considering the fact that toxic effects of PM resided somewhere among several carbonaceous NPs, further experiments should be performed to develop suitable carbonaceous NP mixture models for urban PM.

## Figures and Tables

**Table 1:** Properties of the test particles.

Particles	Primary Particle Size		Hydrodynamic Diameter (μm)		Zeta Potential (mV) pH = 7.4
	Provided by the Manufacturers	FESEM Images	0 h	48 h	
Al_2_O_3_ NPs	10–15 nm	14–60 nm	2.2 ± 0.2	2.4 ± 0.2	−19.5 ± 2.7
TiO_2_ NPs	15–25 nm	15–45 nm	1.4 ± 0.1	4.7 ± 1.2	−23.9 ± 1.4
SiO_2_ NPs	20 nm	13–47 nm	0.8 ± 0.1	1.4 ± 0.1	−21.3 ± 0.5
FeOx NPs	—	0.2–7.6 μm	2.0 ± 0.1	1.9 ± 0.0	−22.5 ± 1.3
MWCNTs	30–50 nm in diameter; 0.5–2.0 μm in length	10–50 nm in diameter	8.5 ± 1.3	8.2 ± 2.1	−21.4 ± 1.2
BCs	30–45 nm	10–60 nm	1.0 ± 0.1	1.1 ± 0.1	−26.6 ± 1.0
GOs	—	2–55 μm	11.9 ± 0.4	28.3 ± 0.7	−18.9 ± 1.3
Graphenes	6–8 nm in thickness; 5 μm in width	1–12 μm	13.2 ± 0.1	11.7 ± 3.0	−27.6 ± 1.6
PM	5.85 μm	1–26 μm	12.6 ± 0.2	14.7 ± 3.1	−18.8 ± 2.3

## Data Availability

“MDPI Research Data Policies” at https://www.mdpi.com/ethics.
